# Exploring theory-based behavioral interventions promoting COVID-19 prevention and healthcare-seeking for migrant worker men in Singapore: a qualitative study

**DOI:** 10.1186/s12889-022-14488-9

**Published:** 2022-11-18

**Authors:** Zoe Jane-Lara Hildon, Chitra Panchapakesan, Md Tahmid Hasan, Nazrana Khaled, Alyssa Yenyi Chan, Shilpi Tripathi, Melvyn Chung Pheng Wong, May O. Lwin, Mark Chen I-Cheng, Kaosar Afsana

**Affiliations:** 1 Saw Swee Hock School of Public Health, National University of Singapore and National University Health System, Tahir Foundation Building, 12 Science Drive 2, Level 09-03J, Singapore, S117549 Singapore; 2 National Centre for infectious Diseases, Ministry of Health, Singapore, Singapore; 3 Institute of High-Performance Computing (IHPC) - A*STAR, Singapore, Singapore; 4 Wee Kim Wee School of Communication and Information, Nanyang Technological University, Singapore, Singapore; 5 BRAC James P Grant School of Public Health, BRAC University, Dhaka, Bangladesh; 6 Raffles Medical Group, Singapore, Singapore

**Keywords:** Migrant workers, COVID-19, Dormitory settings, Prevention measures, Receiving care, Health-seeking, Theory-based intervention design

## Abstract

**Background:**

The first wave of COVID-19 during April to July 2020 in Singapore largely affected the migrant workers living in residential dormitories. A government taskforce working with dormitory operators, employers and non-government agencies came together to deliver behavioral interventions and health care services for migrant worker as dorms were imposed movement restrictions. To fill the research gap in understanding movement restriction experiences of migrant workers, this research seeks to describe dormitory contexts and explore behavior change related to both prevention of transmission as well as healthcare seeking for COVID-19 among male migrant workers.

**Methods:**

With social constructivism as the foundation for this study, 23 telephone interviews were conducted with Bangladeshi and Indian migrant workers. A theory-informed, data-driven conceptual framework, characterized by the “Four Ss”: Sensitization, Surveillance, Self-preservation, and Segregation was first generated and later used to frame second-stage, more in-depth, thematic analyses. An effective multipronged approach was documented, persuading migrant workers in our case-study to improve hygiene and follow some safe distancing measures, and adhere to help-seeking when symptomatic.

**Results:**

Rapid collective adaptation was demonstrated; it was propped up by effective harnessing of infrastructure and technology. While technology and digital platforms were central to shaping Sensitization for prevention-related behaviors, interpersonal communication, especially peer-sharing, was key to normalizing and accepting healthcare delivery and norms about healthcare seeking. Interpersonal factors particularly supported successful implementation of case-detection Surveillance, stimulating Self-preserving and acceptance of rules, and was found helpful to those Segregated in recovery facilities. In contrast, encouraging prevention-related behaviors relied more heavily on multiple online-platforms, phone-based e-learning/knowledge testing, e-monitoring of behavior, as well as interpersonal exchanges.

**Conclusion:**

Overall, the findings showed that the conception of the Four Ss helped inform intervention strategies. Anchoring these towards optimal use of technology and harnessing of interpersonal communication for prevention and promotion of healthcare seeking in the planning of future Infectious Disease outbreaks in closed institutional settings is recommended.

## Introduction

### Background

It has long been recognized that it is not unusual for migrant worker communities to find themselves exchanging the pursuit of prospects abroad against systemic health inequalities [1–3]. In Southeast Asia, sub-standard accommodation [4], limited access to healthcare [5–7] and the pervasive threat of job insecurity and debt burdens [8] have been documented. With ever-looming risks and uncertainties brought by the pandemic, migrant communities have faced many new regulations, enforced confinement, and have shown themselves to be especially susceptible to the impact of COVID-19 [3, 4, 9, 10].

In Singapore, migrant workers’ living conditions were not initially conceived to contain the spread of highly infectious airborne diseases, for example social distancing was especially hard to achieve due to dorm setup and population density [11]. Just 2 weeks after the first imported case was detected on 08 Feb 2020, COVID-19 cases among the migrant workers’ dormitories were identified [12]. Dormitory lockdowns were part of a wider set of community restrictions termed the “Circuit Breaker” (CB) which came into effect on the 7th of April 2020.

#### Dormitory settings

All forty-three purpose-built dormitories in Singapore, which is an urban city state, accommodating approximately 320,000 foreign migrant workers, were put on lockdown from April to August 2020. Residents were mostly males from South Asia, largely employed in low-wage construction, marine, or service sectors [4]. By early May, COVID-19 had rapidly spread in dormitory settings with 17,758 of confirmed COVID-19 cases identified among migrant workers, constituting 88% of all nationally confirmed cases (*n* = 20,198) [4]. Safe management measures were rapidly conceived to manage the outbreak.

Residents were mass-tested, and confirmed infected cases were segregated to the designated areas within the dormitories, government restructured hospitals and community care facilities before returning to their place of residence after treatment and recovery. Most suffered mild symptoms with very minimal deaths, likely to be due to younger aged populations compared to local populations, as well as rapid mitigation responses described below. Dormitories were declared cleared of infection by August 2020. Nevertheless, some movement restrictions remained in place for some time, though recently (November 2021) these were near fully lifted [13].

#### Forward assurance and support teams (FAST)

Dormitory mitigation interventions were undertaken by the Forward Assurance and Support Teams, or “FAST” teams, composed of the Ministry of Manpower, Singapore Armed Forces and Singapore Police Force who worked with dorm operators and also Non-Government Organizations (NGOs) aided by the migrant worker employers [14–18]. These groups worked together to provide food and necessities, and promoting hygiene practices through intensive communication campaigns. Behavioral preventative measures related to environmental and personal protection were quickly unfolded under this and associated initiatives. The Ministry of Manpower worked with employers to ensure workers were paid a basic salary and remittance services to send money home were in reach [14]. Healthcare providers from the public and private sectors were seconded for outbreak mitigation to help with testing, triage, and treatment of dormitory residents [19].

#### Aim and objectives

Given the abundance of behavioral and health services mitigation measures put in place for COVID-19 outbreak in migrant worker settings, we sought to understand the experiences of these from Bangladeshi and Indian migrant workers in one dorm as a case study. The study objectives are to explore:

1. The context of dormitory accommodation during the lockdown;
2. Prevention and behavior change related to reducing transmission, e.g., hygiene and environmental cleaning and hand washing, or safe distancing, including mask-wearing and spacing in groups when possible and so forth;
3. Experiences of receiving care and healthcare-seeking, e.g., testing, going to the doctor and so forth.

## Methods

### Study overview

This case-study is based on the qualitative component of a larger dataset consisting of a survey and telephone interviews undertaken from August–September 2020. Data were collected remotely via a self-completion survey (administered in English, Bengali, Tamil, and Hindi) using the KNOW Application, being used to connect with the migrant workers inside dormitories at the time of the study. The KNOW App is still in use and available for use in closed systems communications in frontline operations, e.g. training and supporting implementation of routine tasks, checklists and data collection (see: KNOW App). Twenty-three participants working in construction were selected from the survey, recruited via telephone, and agreed to be interviewed. It was judged that the objectives of the current paper could be best answered from interview data alone, which forms the basis of the current qualitative analyses. We reported the study methods following the Consolidated Criteria for Reporting Qualitative research (COREQ) checklist [20].

### Research team and reflexivity

The research team consisted of a senior post-doctoral social scientist with qualitative and mixed methods expertise (ZH) who led the study and oversaw the data collection and analysis. The data collection team and analysts consisted of four graduate and post-graduate level public health researchers (TH, NK, CP, ST) all of whom had experience of qualitative research. ST, a middle-aged female researcher, was the key point of contact point and liaison for recruiting and supported the interview process. CP carried out interviews in Tamil, while NK and TH undertook the Bengali interviews. All data collectors were of Indian or Bangladeshi decent. The two interviewers were female, one was male, all were in their late twenties to mid-thirties.

Participants had no prior relationship with interviewers. Rapport-building was initiated over telephone calls and on WhatsApp whilst arranging the interviews. Interviewers shared about themselves and reasons for the study during recruitment, all were public health researchers with interests in the social determinants of health and health inequalities. Even though most participants had a good grasp of English, all interviews were carried out in participants’ native languages, which was seen to further enhance rapport and contribute to trust building. Moreover, total anonymity was emphasized to interviewees.

### Study design

This study is rooted in social constructivism [21–23], taking the stance that meaning making, language (both verbal, non-verbal and symbolic) and processing of these forms the basis of human behavior. Living in closed, densely populated quarters with inevitable group interactions [24], ongoing and sometimes “forced” communication under conditions of intense institutional surveillance will have formed the basis of health behavior experiences of migrant workers under lockdown [25].

At the time of the study, communications on how to behave and begin to understand living with COVID-19 would have been overwhelming. The onslaught of these would have unfolded through interpersonal channels, digital platforms, and multiple competing mass media sources. This context and theoretical underpinnings inform the coding framework described below. We sought to use a purposive sampling strategy selecting a range of ages and the most prominent migrant worker nationalities, Indian and Bangladeshi, within the chosen dorm. See sampling grid, Table 1. Further details on socio-demographics are listed in Table 2 (Results section).

**Table 1:** Sampling grid for selection by variation in age and nationality (*n* = 23), with characteristics reported in aggregate^a^

Sampling by age		Pseudonym	Marital status	Income-range
*Bangladeshi*	Younger: 21–29 years of age	Zafar	*2x single* *2x married*	*Average income is under s$549 per month*
		Rana		
		Habib		
		Rony		
*Indian*		Pandian	*All single*	*Average income is under s$874 per month*
		Murugan		
		Ganesh		
		Natrajan		
*Bangladeshi*	Middle age-band: 30–35 years of age	Amitava	*1x single* *3x married*	*Average income is under s$749 per month*
		Shurid		
		Manik		
		Sakib		
*Indian*		Aravind	*All married*	*Average income is under s$999 per month*
		Mahesh		
		*NA*^b^		
		*NA*		
*Bangladeshi*	Older: 36 years of age or more	Kamal	*All married*	*Average income is under s$949 per month*
		Ijaz		
		Omi		
		Hashem		
*Indian*		Mani	*All married*	*Average income is under s$1149 per month*
		Rachandranan		
		Ramesh		
		Raghu		
		Vishnu		

^a^We have assigned pseudonyms which will be the main reporting criteria tagged to quotes to ensure we preserve anonymity while accurately reporting findings

^b^NA refers to not applicable as unable to recruit

**Table 2:** Description of interview participants

	Sociodemographic information	
Variables	Categories	Interviewees (*N* = 23)
*Age*	21 to 29 years old	8
	30 to 37 years old	11
	38 to 47 years old	4
*Nationality*	Bangladeshi	12
	Indian	11
*Income range*	Less than $500 per month	4
	$500 to $749 per month	10
	More than $750 per month	9
*Marital Status*	Single	7
	Married/ divorced	16
*Highest education level*	Formal education up to high school only	15
	Tertiary education (vocational/ polytechnic/ pre-university, undergraduate)	8
*Time spent away from home country*	Less than 2 years	3
	More than 2 years	20

Interviews were conducted online through an audio call, mostly one-on-one and in some instances with a presence of another team member as note-taker. Interviewees were asked to find a quiet place where they couldn’t be overheard, mostly this was in a secluded corner of shared accommodation inside the dormitory that consisted of sleeping, cooking and bathroom facilities for up to 12 men. The interviews lasted up to 1 h. No repeat interviews were conducted. All interviews were audio recorded with the interviewee’s informed consent.

Participants were asked to narrate their experiences of changes in the dorm since the lockdown, including a more semi-structured component on mental health, reported elsewhere. Narratives quickly focused on prevention-related behavior and healthcare under lockdown conditions [26], and these data and the data explaining the backdrop to how behaviors were formed are the basis of the current analyses. This includes unpacking narratives relating to life in the dorm, fear and worries about COVID-19, views on the best ways to fight the virus, receiving medical care, hygiene and preventative interventions in the dorm and take away messages. The interview guide, see Supplementary file 1, was refined after the initial application and judged fit for purpose, though it was not formally piloted.

Interview data were extracted by adapting the “expanded notes”, and scribing approach [27, 28]. Such approaches rely on detailed summaries of interview data, directly translated into English in the third person, while also extracting salient verbatim, first-person quotes. Data extractions were undertaken by the interviewers within 24 hours using the audio recordings and field notes. Largely due to ethical constraints and to ensure preservation of anonymity, we were unable to recontact participants for member-checking of extracted data and interpretations.

### Analysis and findings

We followed a more inductive approach adapted from Braun and Clark’s Reflexive Thematic Analysis [29]. Analysts (CP, MTH and ZH) first familiarized themselves with the segments of expanded notes relevant to the current objectives. Open coding was initially undertaken to reflexively build a first level of analysis. From this an initial coding, a theory-informed set of broad constructs pertinent to framing both prevention as well as experiences of receiving care and health-seeking, were identified and agreed. These consisted of the “four Ss”, as listed below. In a second stage of coding these constructs were explored more deeply and used as a conceptual framework to guide refining of codes connected to: (1) Sensitization; (2) Surveillance; (3) Self-preservation; and (4) Segregation.

The selection of the constructs used to frame analyses was data driven as well as informed by Erving Goffman’s treatise and recent expansion of Total Institutions [24, 30] and Michel Foucault’s conception of the panopticon effect [25]. Total institution theory was seen as relevant because it defines closed systems where formal routines and structures are enacted, sensitizing those segregated within the system to shared norms and practices. Foucault’s conception of the panopticon was seen to reflect an observed internalizing of behavior prompted by an emphasis on surveillance and rule following, which is ultimately connected – among other factors – to self-preservation. Such features were seen as central to contemporary migrant worker’s housing conditions during national lockdown in the present setting.

Coders divided analyses according to objectives, shared and reviewed findings and reached a consensus on the meaning of the data. Data were managed using Atlas.ti 9. Saturation was quickly achieved in early stages of coding in relation to shared understanding of prevention-related behaviors, messaging, and the internalizing of these as well as on perspectives and experiences of healthcare. Less depth was achieved on the latter, partly because only two experiences of two COVID positive cases were encountered.

**Fig. 1:**
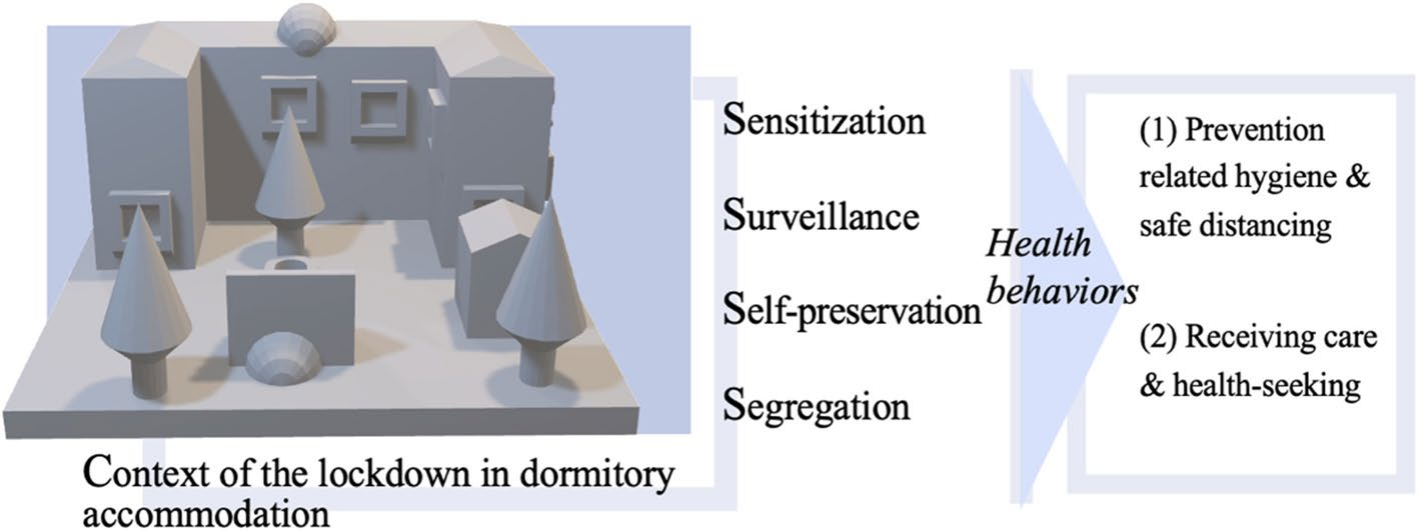
Conceptual data-driven framework, comprising of the four Ss, supported by dormitory contexts, explaining health behaviors

The findings for each objective are addressed in turn, guided by the proposed theory driven conceptual framework [31], summarized Fig. 1. Major themes are reported in *bolded italics* and supporting sub-themes narrated to address context of daily life in the dorm and the four Ss. We have assigned Pseudonyms (Table 1) which is the main reporting criteria tagged to quotes to ensure anonymity.

### Ethical review

The study was conducted with approvals from the Departmental Ethical Review Committee (DERC) institutional review board at Saw Swee Hock School of Public Health, National University of Singapore.

## Results

### Sample description

The characteristics of the all-male participants working in the construction sector (*n* = 23) are described in Table 2. All participants were legally employed and in possession of valid work passes. We achieved a near equal distribution of Indian and Bangladeshi nationalities. The mean age of the participants was 32, ranging from 21 to 47 years of age. More than half of the participants were married (*n* = 16) and reported their education level as junior high or below (*n* = 15). Most (*n* = 14) earned less than S$749 per month. Only three participants had been in Singapore for less than 2 years.

### Objective 1: context of the lockdown in dormitory accommodation

#### Life in the dorm

The context of the dorm during the study period was characterised largely by *rapid collective adaptation*. Data showed how initial panic yielded to widespread regaining of composure and finally acceptance, especially as restrictions eased. For instance:

“We all were scared [ … ] when they told me that they would keep me in isolation inside the dorm, I felt lost, I panicked. It was a difficult time. I thought to myself, would they deport me if I tested positive? I knew the steps that the Government was taking, but I could not believe it. [At first] I thought they would not follow through on them.”

- Rony, younger Bangladeshi

“In my room, three people got positive. By that time, it was common, so we didn’t have any fear. We just thought that the test is positive. If it was during the starting time, we would have gotten scared, by that time, a lot of people were tested positive … No one was afraid about it.”

- Aravind, middle age-band Indian

“I feel that COVID-19 is not dangerous, but a normal disease. Now [the lockdown] is over. We should not be afraid of this. By maintaining protection measures, we can protect ourselves.”

- Raghu, older Indian

To begin with, procedures around isolation, testing and treatment were constantly shifting, adding to the initial fears. Mass swabbing was initiated. Suspected COVID-19 cases were initially isolated to another room within the dormitory. Once their test results were confirmed, residents were either sent back to their room if negative, or if positive, moved to treatment centers.

Meanwhile, it was widely shared that extensive cleaning and hygiene precautions were swiftly undertaken and enabled by the Dorm Management as well as employers:

“From the dorm, they did many things. They cleaned the whole dorm, sanitized all areas using spray. The bathroom and toilets are outside the room [ … ]. The dorm authorities clean the bathrooms and toilets. They have also kept dustbin in the corridor, and we eat our food and throw the waste in the dustbin [ … ]. The dorm was clean even before, before COVID we could say around 75% and now it’s clean 100%.”

- Vishnu, older Indian

“In our workplace, they spray disinfectants on us before we enter, and after we leave, they again spray disinfectants. Apart from that, they advised us to use hand sanitizers before and after using any tool, and to wear gloves at all times.”

- Rana, younger Bangladeshi

Appreciation for expediting set-up of treatment facilities within the dorm and government provision of food and basic necessities were at times emotively expressed:

“We got our free food, but we were not sure who was the provider, how would we pay for it, and other issues. When the government explained to us [it was free], we were very surprised [ … ]. I don't think I would get any better treatment if I were in Bangladesh [ … ]. Certainly, I would not get half of the facilities I am getting here, despite being only a worker. [ … ] The Singaporean government announced that we [the workers] would get all the facilities of a Singaporean citizen, and it meant a lot. I will never forget how they cared for us in the most crucial time of our lives.”

- Rony, younger Bangladeshi

“They [dorm management] did well [delivering testing]. They called us serial-wise and didn't make anybody wait for too long [ … ]. The results were returned in 2-3 days through an App.”

- Hashem, older Indian

**Fig. 2:**
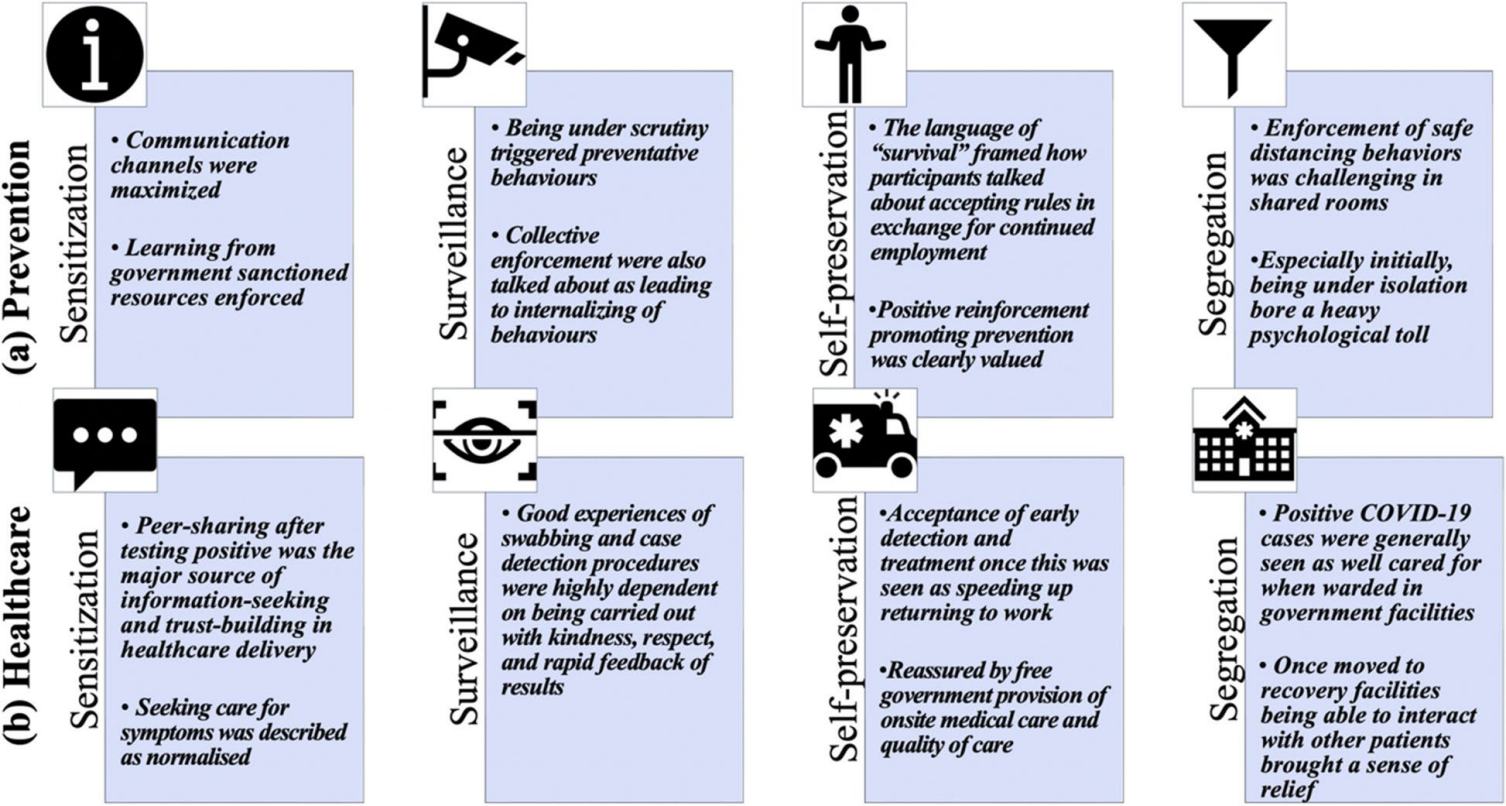
Mapping themes to conceptual data-driven framework, explaining prevention and healthcare-related behaviors in the dormitory contexts

The *enabling role of technology and infrastructure* in support of efficient unfolding of logistics was also notable. As illustrated above, digital communication platforms were stepped up by government entities. Existing surveillance infrastructure, e.g., dorm cameras and policing were harnessed and enhanced to mitigate COVID-19. Such findings are further explored in forthcoming analyses, for which major themes are summarized in Figs. 2.

### Objective 2: prevention and behavior change related to reducing transmission

#### Sensitization for prevention

Relating to sensitization for raising awareness of preventative behaviors, *communication channels were maximized* and *learning from government sanctioned resources enforced.* Participants spoke about actively searching about information on COVID-19 through government bulletins, local and home country news sites, and social media. “Probashi”, a Facebook page for Bangladeshi migrants living in Singapore was particularly highlighted, information on this page was trusted and believed to be managed by doctors. Though lay beliefs about immunity building spices and hot drinks were sometimes shared, accurate symptom recognition and prevention knowledge was common, as illustrated:

“The government, my company, my friends and family, everybody keeps saying how to prevent COVID-19. They are saying the same things, to wear masks, gloves, to maintain distance, to wash hand and face etcetera.”

- Sakib, middle age-band Bangladeshi

The interviewees shared that knowledge was acquired via government sources because migrant workers were required to pass a COVID-19 awareness test, some also qualified as safe distancing officers:

“We had to take an exam via our mobile around one month after lockdown. The questions were like how to wear masks, how to remove it, that the blue side should face out and so on. Our company told us that if we could not pass the exam, then we could not resume working, we must pass it, and to do so, we must listen to their briefings [ … ], they told us about COVID, about masks and many other things.”

- Omi, older Bangladeshi

“I took an online exam on COVID-19 and I have also finished a safe distancing officer course. I have read a lot of books about how to handle yourself in public places.”

-Vishnu, older Indian

Even in these early days, information on availability of vaccines was sought by some, hoping this will help get things “back to normal”:

“No one knows when the vaccine would arrive, or when everything would go back to normal. The authorities are also silent. They should tell us a probable time at least."

- Rana, younger Bangladeshi

“The biggest tension in the beginning was what would happen if I got affected. So far, there is still no vaccine for the virus and because of that, it is critical. That was a source of tension.”

- Shurid, middle age-band Bangladeshi

“If the vaccine came, then it would be best. Until then, it is best to stay alert for one's own safety.”

- Hashem, older Bangladeshi

“Till vaccine comes [ … ], we must follow all the rules.”

- Raghu, older Indian

Overall, digital channels were used with unprecedented intensity. Inferences or statements such as “I spent most of the lockdown time on my phone” were not unusual. Interviewees displayed early sensitization and acceptance for COVID-19 vaccines, concomitant to a thirst for knowledge on how to prevent transmission. This reflected the drive for self-preservation and desire to return to work but also the drive to be free of constant surveillance.

#### Surveillance for prevention

Surveillance for prevention took the form of monitoring of movements, behaviors / rule following. Certainly, *being under scrutiny triggered preventative behaviors*, though this does beg the question of whether these can be sustained. As mentioned above, government surveillance began with monitoring knowledge of how to prevent the spread of the virus. Closed-circuit television (CCTV), common in residential areas of Singapore for security reasons, and constant security patrolling were the norm in the dormitory:

“There are CCTVs in the hallway, security guards, dorm authority, and even sometimes there are police inside the dorm. You cannot get away from them, they will catch you, if you are found without wearing masks or breaking any safety protocols.”

- Kamal, older Bangladeshi

“The dorm was clean even otherwise, but they [the dorm operators] kept it clean during covid as well. They would do their work perfectly. There are cameras everywhere so if someone messes, they will be fined immediately as well. So everyone else will keep it clean as well.”

- Mahesh, middle age-band India

“Policing” inside the dorm by onsite officers and fines for not wearing masks or social distancing, plus surprise Ministry of Manpower inspections were mentioned. Older participants spoke about these conditions being reinforced by inspections, peer monitoring and eventually teamwork:

“As a safe distancing officer, when you see a person doing something wrong, you have to explain and correct them. Explain to them don’t do this as the disease is spreading because of actions like this. It is my duty.”

-Vishnu, older Indian

“We look after one another, as we do not want to get one of our colleagues fined. So, if someone managed to slip through all of this, anyone of us would tell him to go back and bring his mask [ … ]. It's for him, as well as our safety."

- Kamal, older Bangladeshi

“We are seven people sharing dorm room and hall which is quite spacious. We all divided our work that someone clean the bathroom, someone clean the bedroom and someone sweep the floor. Someone will go dormitory canteen and bring the food. And in the evening we would iron our clothes.”

- Ramesh, older Indian

These forms of *collective enforcement were also talked about as leading to internalizing of behaviors*, such as new shared, observed habits or new routines:

“Before lock down we used to clean twice a week, and during lock down we used to clean whenever we feel like it as we are home all the time and we are sitting without any work and will decide to clean suddenly to pass time. Sometimes we clean it daily or every other day.”

- Mahesh, middle age-band Indian

“Now, we clean it daily as we are in the room all day [ … ]. Previously, a lot of people won’t even wash their hands before eating food for sure, 100%. Now they follow it nicely.”

- Mani, older Indian

Other forms of government surveillance such as contact tracing, i.e., tracking devices or use of TraceTogether App were accepted as a necessary part of “surviving” the pandemic and being in Singapore. Further digital monitoring, especially of temperature-taking were occasionally supported to reduce the hassle and problems of self-reporting:

"When the government provided us with the thermometers, some of my roommates did not know how to operate it. They often held the thermometer by the mercury-end, and reported the number after measurement. Before the pandemic, we used to help them with the measurement if one of them needed, but now it is not possible. If the government provided us with a wrist device, it would be great. It would greatly reduce the hassle of frequently measuring our temperatures, and at the same time such errors would not occur."

- Rony, younger Bangladeshi

A further few commented that monitoring prevention enforcements would be what made some countries able to overcome the effects of COVID-19, and they also expressed that everyone needed to follow these measures for them to work effectively:

“If and when government sets rule we have to follow that, it will not be just for me it will be for everyone. I will definitely follow rules.”

- Rachandranan, older Indian

“We can’t do this alone. We all have to follow [the rules]. If one person follows and another doesn’t, it will not work. This has to be done as teamwork. Only then it will work.”

- Mani, older Indian

“The government has advised us to do many things, and in all honesty I believe all these things are important. [ … ] The countries that took the rules of wearing masks, social distancing seriously have survived this pandemic”

- Rony, younger Bangladeshi

Overall, strict measures taken by the government were viewed positively by some against the broader context and global response.

#### Self-preservation and prevention

Attitudes that lent into rather than away from following rules were underpinned by a desire for self-preservation. *The language of “survival” framed how participants talked about accepting rules in exchange for continued employment*. Fear as a motivator for uptake of prevention-related behaviors was unanimously referenced. This was primarily supported by the need to keep oneself well, working and providing for family living at home:

“This pandemic ate up all of my family's savings, the situation is so bad that if I lose my job and deported to Bangladesh, we may end up starving [ … ]. Things won't go this bad if only my cousin and one of his child were not in ICU [intensive care unit] for almost a month. We had to share the burden of paying the bills, and just before that my father had a heart surgery.”

- Rana, younger Bangladeshi

Fear of loss of income due to deportation or falling sick, or for whatever reason not being able to help provide – while having the obligation to do so – i.e., covering medical bills or family’s living costs, often laid on further because of the pandemic, was pervasive. While under such conditions the imposed rules were often well tolerated, *positive reinforcement promoting prevention was clearly valued*, although this was rarer than top-down imposition of rule following:

“There was an online competition where we had to upload the pictures of our room, washroom, kitchen, and if they are satisfied then they would give us a 50 Singaporean dollar voucher. It was a nice idea, but they only did it once.”

- Sakib, middle age-band Bangladeshi

In addition, while strict imposing of rule following has obvious direct benefits for stemming transmission, fear of upsetting authorities and possible retribution from this also stopped people wanting to communicate problems in the dorm. Complaints were fielded through employers, making their role pivotal to ensuring dignity was preserved and basic needs met. Nevertheless, there was also evidence that relationships with dorm operators grew over time, often yielding to comradery formed from the shared desire to bring back normality.

#### Segregation for prevention

Blockwide lockdown *enforcement of safe distancing behaviors was challenging in shared rooms*, especially within those with many occupants, though participants reported trying:

“This is a small room and we are usually nine to twelve people so it's not always possible to maintain one metre distance between ourselves. But we try to do it as much as possible.”

- Ijaz, older Bangladeshi

“It isn't always possible to [maintain social distance] in the rooms. We try to do it when having our meals, but when we pray together, we don't always manage. Though when going to sleep everyone goes to their own bed, nobody sits on or sleeps on another bed anymore.”

- Manik, middle age-band Bangladeshi

Prayer time was especially mentioned as a time that people did not necessarily distance. This was considered important bonding time that helped protect psychological health. *Especially initially, being under isolation bore a heavy psychological toll*.

### Objective 3: experiences of receiving care and healthcare-seeking

#### Sensitization and healthcare

Initially there was little information on what would happen if tested positive. Testifying to the central role of interpersonal communication, *peer-sharing after testing positive was the major source of information-seeking and trust*-*building in healthcare delivery*:

“I was probably one of the first to be tested COVID-19 positive from our room. When I did not return that day after swab testing, my roommates kept calling me. I told them that the dorm authority was suspecting me as positive, and kept me in isolation. They seemed very tense [ … ]. We used to video call each other, and I showed them how I am, where they have kept me and how things are. First, at the isolation room in the dorm, and then in [facility name]. They were relieved that I am in a good condition and under excellent care in a good place.”

- Rony, younger Bangladeshi

This informal interpersonal channel of communication on sharing about recovery was widely described even though only two of our interview sample was treated for COVID-19; migrant workers were in constant communication, and constantly advising one another. Due to this, as time went on, *seeking care for symptoms was described as normalised* by some:

"When [my elder brother] suddenly noticed that he had lost his sense of smell and had a cough. He was afraid and shared this with me and I told him to get tested. After testing, when the result came positive, the dorm management sent him to hospital.”

- Amitava, middle age-band Bangladeshi

This finding demonstrates the power of informal networks and communication channels.

#### Surveillance and healthcare

Surveillance to detect and isolate infected cases took the form of serology testing, relentless swabbing, as well as daily self-monitoring and e-recording of temperature. *Good experiences of swabbing and case detection procedures were highly dependent on being carried out with kindness, respect, and rapid feedback of results*:

“The procedure [swab] felt scary at the beginning, but the doctors were really good. I still remember one joked it was like putting a lollipop mistakenly into the nostril. It was very funny and really helped me relax. [ … ]. I was swabbed probably five to six times and my blood was tested once. In the beginning, we used to get the result in the third or fourth day [ … ], on paper. But around July we began getting our results through the App. One could find everything related to COVID in that App. It was a wonderful initiative.”

- Omi, older Bangladeshi

“They won’t tell us about results, not if its negative. [ … ] After the swab test we waited for them to inform the results but they didn’t [ … ]; as we spent a lot of time on the phone, we figured it out ourselves that we can find the results in this App. Now we just need to scan the permit and we can find out the results.”

- Natrajan, younger Indian

“Around five people in my room were scared as that they had COVID-19 when they went for testing. They even packed their stuff thinking security won’t give them enough time to pack things if they turn out to be COVID-19 positive.”

- Mani, older Indian

As repeatedly illustrated, not receiving, or knowing where to find COVID-19 testing results caused anxiety. This was common in the beginning as systems were being set up, future such care delivery should seek to avoid this. Recovered cases who did not initially know they were sick due to being a-symptomatic were identified through serology, communicating these results in turn helped to ease fear, and diffuse dorm lockdown tensions.

#### Self-preservation and healthcare

Overall**,** we observed *acceptance of early detection and treatment once this was seen as speeding up returning to work*:

“If we had any issue they would check us immediately. It was a minor issue, they will treat us and send us back. If it was serious, then there were also two to three ambulance for bringing us to hospitals. They took care us, gave us quality treatment and made sure that no issues happened [ … ]. They took preventive action clearly.”

- Vishnu, older Indian

“If I can remain well, then my family will also remain well because I will be able to work and send them money. Whatever steps the government takes must be for our own good; the Singapore government is different from that in other countries, they only focus on the good things for the people.”

- Habib, younger Bangladeshi

Related to this, as noted earlier, interest in receiving the vaccine was already expressed at this early stage of the pandemic. The study participants also expressed being *reassured by free government provision of onsite medical care and quality of care*:

“[At the outset] I was thinking and making up situations in my head. I thought if they deported me back home, at least I would be able to die in the presence of my family, it would be better to die there. But if they kept me here, treated me, *and later charged me for that* [emphasis our own], I would have no other way but to commit suicide. So, I made up my mind that I would tell them to deport me instead if they want to do that [charge for treatment and other things].”

- Rony, younger Bangladeshi

The fact that care was free at the point of delivery, allowing participants to keep healthy and working was valued and protective.

#### Segregation and healthcare

*Positive COVID-19 cases were generally seen as well cared for when warded in government facilities*. Healthcare was swiftly imposed as was quarantining until recovery (two negative COVID-19 tests in a row). Though there remained uncertainty around these processes, especially around length of stay, the provision of equitable, quality care was also frequently mentioned, e.g.:

“[Those who tested positive] were taken to the hospital, and then kept [various places mentioned] in temporary camps. Different people stayed there for different amounts of time - some for 15 days, others for 45 days, even two to three months. Then, they were brought back to the dormitory. They set up tents within the premises and some [recovering people] are kept in separate blocks.”

- Hashem, older Bangladeshi

“I saw that if anyone had a simple fever, cough or similar issues, they immediately transferred them to the hospital, provided proper medical care and treatment. If anyone had any additional health issues, they also treated them. My friends told me that the hospitals were very good, they took great care of them, they never faced any kind of discrimination whatsoever for being a [migrant] worker or for not being a citizen.”

- Omi, middle age-band Bangladeshi

Peer-sharing is once again highlighted, positive and ongoing interpersonal communications were widely implied as contributing to a stable, and ultimately normatively accepted detection-containment protocol. Those who did test positive mentioned that *once moved to recovery facilities being able to interact with other patients brought a sense of relief*. They could see how other patients were doing and were reassured that most were doing well. In addition, in some recovery facilities, it was shared that compared to the dormitory, residents in recovery were able to move around more freely and interact with one another, e.g. by volunteering to help run daily activities, which was described as appreciated.

## Discussion

During the height of dormitory lockdowns, it was identified that while technology and digital platforms were central to shaping sensitization for prevention behaviors, interpersonal communication, particularly peer-sharing, was key to normalizing and acceptance of healthcare delivery and norms relating to healthcare seeking. In fact, interpersonal factors were observed as central to healthcare planning, i.e., successful implementation of case-detection surveillance, stimulating self-preserving acceptance of rules, and helpful to those segregated in recovery facilities.

In contrast, prevention behaviors relied heavily on online platforms, phone-based e-learning / testing, being watched on camera, as well as interpersonal exchanges. Though, in addition, successful implementation of app-based communicating of testing results was also achieved and demonstrated to be of central value in the healthcare delivery to migrant workers living under outbreak conditions, or under movement restrictions.

The four Ss were observed to be independently and rather matter-of-factly described. They are also interconnected, e.g., a high surveillance setting can lead to self-policing and internalizing of ultimately self-preservation driven habits such as handwashing or healthcare seeking when symptomatic, *even when these things are no longer imposed*. The suggested framework is data driven, elements of which are reflected in existing policy and planning for migrant workers in holding centers or in humanitarian settings [32, 33] and facing high transmission outbreaks, e.g., cholera, typhoid etc, as well as viral, airborne outbreaks.

These existing studies confirm the importance of addressing: segregation, for instances barriers to physical distancing, even physical infrastructure as a key intervention strategy; sensitization, e.g., risk communication alongside meaningful community engagement, trust building; also enabling surveillance through appropriate monitoring. Yet, underlying agency [34], or will and desire for self-preservation to be able to keep productive is not captured. The current study found that participants did not identify themselves as passive victims of the circumstances, though they were living under tightly regulated conditions. Indeed, most respondents expressed being wilfully compliant, and in many cases grateful. A few even had spoken about how they believed that countries who were most aggressive in containing COVID-19 would be the ones to fair the best in the long run.

This is supported by evidence that shows humans are more compliant to social distancing pandemic rules in the face of uncertainties and perceived higher risk [35]. Risks for the migrant workers in the present study were multifaceted – socioeconomic as well as, initially at least, tied to fear of being infected. This was combined with uncertainty about the undetermined period of lockdown and other worries. Nevertheless, psychological effects of being physically cut off, amongst others concerns, though mentioned by many migrants, were also quickly mitigated by their own adaptive capability as well as solidarity within their communities. Such effects are fully reported elsewhere [26].

Overall, our participants indicated they were highly adaptive and attuned to protective interventions that would aid a return to normality, even demonstrating high desire for vaccines before these were available. Tapping into this aspect of human capability, using positive rather than enforcing strategies will be key to long-lasting behavior change.

### Strengths and limitations

The team faced challenges with e-interviewing. For instance, some respondents initially hesitated to open-up about their lives due to not meeting the researchers in person, researchers’ identities were confirmed by showing identity cards by video call and re-iterating information and assurances from the Participant Information Sheet and Consent (PISC) taking. Though we acknowledge the risk of social-desirability given the sensitivity and high tensions as data were being collected, rapport-building by researchers from participants’ native countries, conducting interviews in participants native language and stressing anonymity were key to encouraging open sharing.

Though limited to one dormitory setting, and a case study of Bangladeshi and Indian migrant workers only, the study provides an in-depth analysis of what was at the time of the study a hard-to-reach population. Subsequent studies should consider the transferability of our findings across other migrant worker nationalities and in international settings beyond Singapore.

## Conclusion

Based on research findings to improve prevention and health-seeking behaviors we broadly recommend the use of the four Ss for planning purposes in closed settings experiencing highly transmittable outbreaks. More specifically, we recommend consistent positive framing to engaging communities as well as doing so through peer ambassadors and leveraging benefits of self-preservation to promote adherence to sensitization messages. Once knowledge is internalized, accepted, and habitually practiced by the individuals, and communities, the health practices will be more likely to be sustained without external surveillance or imposed segregation.

## Data Availability

The datasets generated and/or analysed during the current study are not publicly available due to the sensitive nature of the study and to preserve anonymity as per the study team’s commitment during institutional ethical review, but are available from the corresponding author on reasonable request.

## Abbreviations

CB: “Circuit Breaker”
NGOs: Non-government Organizations
COREQ: Consolidated Criteria for Reporting Qualitative Research
PISC: Participant Information Sheet and Consent
